# External treatment of traditional Chinese medicine for constipation after thoracolumbar compression fractures: A protocol for systematic review and meta-analysis

**DOI:** 10.1097/MD.0000000000027110

**Published:** 2021-09-03

**Authors:** Xinru Liu, Yilan Wang, Qing Ye, Yiming Sun, Jie Yang, Yu Dai, Quan Wen

**Affiliations:** a Traditional Chinese Medicine Department, Chengdu Eighth People's Hospital (Geriatric Hospital of Chengdu Medical College), Chengdu, China; b Clinical College, Chengdu University of Traditional Chinese Medicine, Chengdu, China.

**Keywords:** constipation, external treatment of traditional Chinese medicine, meta-analysis, protocol, thoracolumbar compression fractures

## Abstract

**Background:**

Constipation is one of the common complications of thoracolumbar compression fractures, which seriously affects the quality of life and increases pain of patients. External treatment of traditional Chinese medicine (TCM) has been widely used clinically for constipation after thoracolumbar compression fractures, but there are no systematic review and meta-analysis of its efficacy. Therefore, we will conduct this study to systematically evaluate the clinical effects of external treatment of TCM for patients with constipation after thoracolumbar compression fractures.

**Methods:**

We will search the following electronic databases: PubMed, Embase, Cochrane Library, Web of Science, China National Knowledge Infastructure, Chinese Biomedical Literatures Database, Chinese Scientific Journal Database, and Wanfang Database. Randomized controlled trials on the treatment of constipation after thoracolumbar compression fractures with external treatment of TCM published from inception to May 2021 will be included in the search scope. The observation group was treated with Simple external treatment of TCM (such as external application of Chinese medicine, Chinese drugs at the acupoint, acupuncture, moxibustion, etc) or external treatment of TCM combined with conventional treatment/nursing of Western medicine, while the control group only was treated by conventional treatment/nursing of Western medicine. After screening literatures, extracting data, and assessing the risk of bias in the included studies, meta-analysis will be performed by Revman 5.3 software.

**Results:**

This study is expected to provide an evidence of the efficacy of external treatment of TCM for constipation after thoracolumbar compression fractures.

**Conclusion:**

The results of this meta-analysis may help provide evidence to determine whether external treatment of TCM can be effective interventions for thoracolumbar compression fractures patients with constipation.

**Trial registration number:**

INPLASY202150005

## 1 Introduction

Thoracolumbar compression fracture is a common disease in the department of orthopedics, accounting for 30% to 60% of spinal fracture.^[1]^ Osteoporotic patients are prone to vertebral compression fractures,^[2]^ so the incidence rate of this disease in elderly people is much higher than that in young people. Patients must stay in bed due to pain stimulation and a series of pathophysiological changes occur after thoracolumbar compression fractures, both of which will induce abdominal distension, constipation and other gastrointestinal disorders.^[3]^ Research has shown that the proportion of patients with thoracolumbar compression fractures complicated with constipation is more than 80%.^[4]^ If not solved in time, it will seriously affect the quality of life.

As for constipation, Western medicine currently focuses on symptomatic treatment, adopting treatment methods such as increasing gastrointestinal motility, antispasmodic and pain relief, catharsis, and cleaning enema, which may be prone to repeated illness, drug resistance, and drug side effects.^[5]^ The choice of treatments for constipation after thoracolumbar compression fractures remains a challenging clinical problem.

The external treatment of traditional Chinese medicine (TCM) is a unique traditional treatment method with a long history in China. It is simple, safe, effective, and can be applicable in various ways. This method has been widely used in clinical treatment of constipation.

It has been pointed out in meta-analysis that external treatment of TCM can significantly improve constipation symptoms of Parkinson disease and reduce the incidence of constipation in patients with acute stroke.^[6,7]^ However, whether the evidence is transferable to patients with constipation after thoracolumbar compression fractures remains unclear.

At present, many clinical trials have found that the external treatment of TCM (such as acupuncture, acupoint application, and external application of Chinese medicine, etc) are effective in the treatment of constipation after thoracolumbar compression fractures.^[5,8,9]^ The purpose of this study is to systematically review current available literatures to evaluate the efficacy of external treatment of TCM for constipation after thoracolumbar compression fractures.

## 2 Method

### 2.1 Study registration

This protocol has been registered on INPLASY and the registration number is INPLASY202150005. The protocol report is implemented depending on the Preferred Reporting Items for Systematic Reviews and Meta-Analyses Protocols.^[10]^

### 2.2 Inclusion criteria

#### 2.2.1 Types of study

Randomized controlled trials of all external treatments of TCM for constipation after thoracolumbar compression fractures published in domestic and foreign medicine, whether is blind or not, will be included. Letters, case report, animal studies, observational research, meta-analysis, reviews, and conference articles will be excluded. In addition, studies on constipation after surgical treatment of thoracolumbar compression fractures or the prevention of constipation after thoracolumbar compression fractures will also be excluded.

#### 2.2.2 Types of participants

Patients who were diagnosed with constipation after thoracolumbar compression fractures and treated with external treatment of TCM were included, regardless of age, gender, and course of disease.

#### 2.2.3 Types of interventions

##### 2.2.3.1 Experimental interventions

Simple external treatment of TCM (such as external application of Chinese medicine, Chinese drugs at the acupoint, acupuncture, moxibustion, etc) or external treatment of TCM combined with conventional treatment/nursing of Western medicine will be included in the treatment group. Specific techniques, courses, and frequency are unlimited.

##### 2.2.3.2 Comparator interventions

For control group, patients only were treated by conventional treatment/nursing of Western medicine.

#### 2.2.4 Types of outcomes measures

##### 2.2.4.1 Primary outcomes

The primary outcome is the total effective rate.

##### 2.2.4.2 Secondary outcomes

The secondary outcomes include constipation symptom scores (such as defecation time, defecation interval, effort level, stool characteristics), first defecation time, first exhaust time, quality of life, and adverse events.

### 2.3 Search strategy

#### 2.3.1 Electronic searches

The databases will be searched for randomized controlled trials on the treatment of constipation after thoracolumbar compression fractures with external treatment of TCM by PubMed, Embase, Cochrane Library, Web of Science, China National Knowledge Infrastructure, Chinese Biomedical Literatures Database, Chinese Scientific Journal Database, and Wanfang Database. The search time ranges from the establishment of the database to May 2021.

The search terms will include “External treatment of traditional Chinese medicine” “acupuncture”, “moxibustion”, “electroacupuncture”, “Acupoint application”, “Auricular point”, “Massage”, “thoracolumbar compression fracture”, “constipation”, etc. The search strategy for PubMed is summarized in Table 1.

**Table 1:** Search strategy for PubMed.

Number	Search terms
1	Medicine, Chinese traditional
2	Chinese traditional medicine
3	Chinese medicine, traditional
4	Traditional medicine, Chinese
5	Traditional Chinese medicine
6	TCM
7	Chinese medicine
8	Drugs, Chinese herbal
9	Herbal medicine
10	Medicine, herbal
11	Or 1 to 10
12	External treatment of traditional Chinese medicine
13	External treatment
14	External therapy
15	Or 12 to 14
16	Acupuncture
17	Acupuncture therapy
18	Acupoint application
19	Auricular point
20	Acupoint
21	Electroacupuncture
22	Hot medicated compress
23	Moxibustion
24	Needing
25	Cupping
26	Catgut embedding
27	Retention enema
28	Massage
29	Manipulation
30	Or 16 to 29
31	11 or 15 or 30
32	Randomized controlled trial
33	Controlled trial
34	Clinical trial
35	Randomly
36	Randomized
37	Trial
38	Or 32 to 37
39	Fractures, compression
40	Fracture, compression
41	Compression fracture
42	Compression fractures
43	Thoracolumbar compression fracture
44	Vertebral compression fracture
45	Spinal fractures
46	Spinal fracture
47	Fracture, spinal
48	Fractures, spinal
49	Or 39 to 48
50	Constipation
51	Dyschezia
52	Colonic inertia
53	Gastrointestinal disorder
54	Or 50 to 53
55	31 and 38 and 49 and 54

#### 2.3.2 Searching other resources

Reference lists of primary studies and relevant reviews will be manually searched to identify potential references. We will also conduct a search on the website of ClinicalTrials.gov, WHO International Clinical Trials Registry platform, and Chinese Clinical Trial Registry to avoid missing ongoing or unpublished studies.

### 2.4 Data collection and analysis

#### 2.4.1 Selection of studies

**Figure 1:**
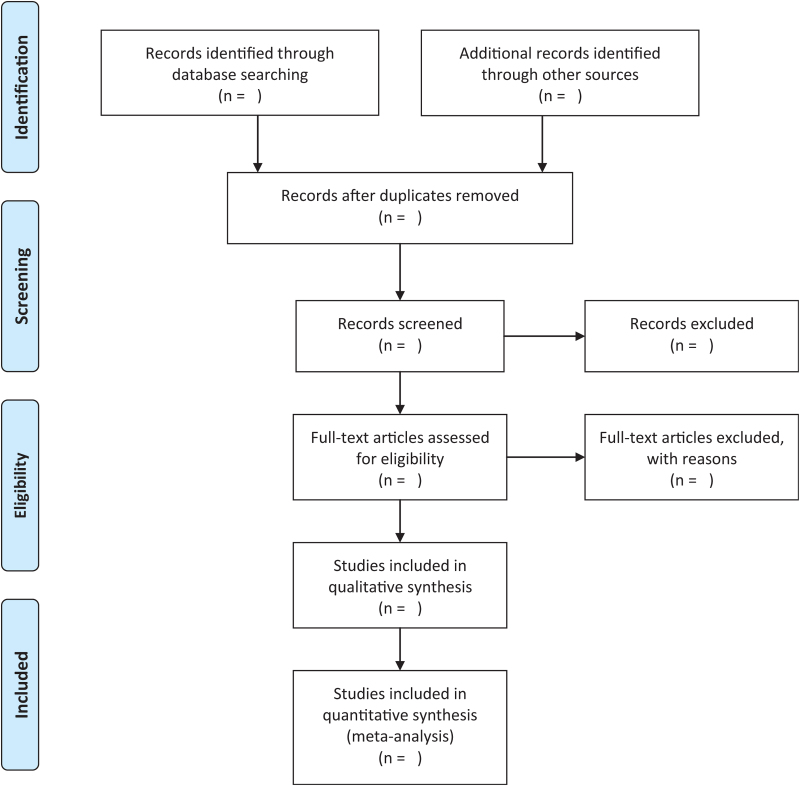
Preferred reporting items for systematic reviews and meta-analyses flow diagram.

All searched literatures will be imported into the Endnote software 9.0 for management and deduplication. Two reviewers will independently scan all retrieved literatures’ title and abstract in strict line with the inclusion and exclusion criteria, then ruled out the unqualified literatures. After that, the reviewers will read the full text of preliminary screening documents in detail to determine final inclusions. Any disagreement will be resolved through consensus or discussion with a third reviewer. A Preferred Reporting Items for Systematic Reviews and Meta-Analyses flow diagram will be used to illustrate the process of research selection in Figure 1.^[11]^

#### 2.4.2 Data extraction and management

Two authors will extract data from the qualified articles. The following information will be extracted from the literatures: title, author, year of publication, participants’ demographic data, sample size, study design, study duration, interventions, main results, adverse events, and other information. Any disagreement will be resolved by consensus or consultation with a third review author.

#### 2.4.3 Assessment of risk of bias in included studies

The Cochrane risk of bias tool will be used to assess 7 aspects of risk of bias, including sequence generation, allocation concealment, blinding of participants and assessors, blinding of outcome assessment, incomplete outcome data, selective reporting, and other bias. We will divide the risk of bias into 3 levels: low risk, high risk, and unclear. This assessment will be conducted independently by 2 reviewers, and any difference in the assessment process will be resolved through consultation with the third reviewer.

#### 2.4.4 Measures of treatment effect

For continuous outcome, we will use mean difference with 95% confidence intervals to measure the treatment effect, and dichotomous data will be analyzed by risk ratio with 95% confidence intervals.

#### 2.4.5 Dealing with missing data

If possible, we will contact the author of the original article for missing data. Only available data will be included in the primary analysis.

#### 2.4.6 Assessment of heterogeneity

χ^2^ test and I^2^ statistic will be used to assess the statistical heterogeneity. It indicates statistically significant heterogeneity when the *P* value <.10 or I^2^ value is more than 50%, and subgroup analyses will be performed to identify possible reasons at same time.

#### 2.4.7 Assessment of reporting biases

If there are more than 10 studies included in a meta-analysis, funnel plot will be performed. And the Egger regression and the Begger tests will be calculated to test the asymmetry of funnel plot.

#### 2.4.8 Data synthesis

Review Manager (RevMan) V.5.3 Software will be used for data synthesis. The random-effects model will be used to synthesize the data if I^2^ > 50% while the fixed-effects model will be used if I^2^ < 50%. If a meta-analysis is not possible, we will provide a narrative summary of the results from individual studies.

#### 2.4.9 Subgroup analysis

When there is a significant heterogeneity in the studies, we will conduct a subgroup analysis based on age and sex, type of intervention, treatment courses, and outcome measurements.

#### 2.4.10 Sensitivity analysis

If possible, a sensitivity analysis will be performed to verify the robustness of the review conclusions by evaluating the impact of methodological quality, sample size, and missing data.

#### 2.4.11 Ethics and dissemination

Ethical approval is unnecessary because no primary data will be collected. The results of this systematic review will provide an effective and reliable basis for external treatment of TCM for constipation after thoracolumbar compression fractures, and will be published in a peer-reviewed scientific journal.

## 3 Discuss

With the advent of aging, thoracolumbar compression fractures are becoming more and more common clinically, and most patients are often accompanied with symptoms of constipation as the disease progresses. Modern medical treatment for this diseases is also limited. Nowadays, external treatment of TCM has been more and more widely used in constipation. We search the clinical research on this disease in recent years and find that the curative effect of external treatment of TCM is significant, and there are no related systematic review and meta-analysis report. Therefore, our study will evaluate the current evidence on the effectiveness of external treatment of TCM to provide more treatment options for patients with constipation after thoracolumbar compression fractures, and encourage more peer experts and doctors to carryout as much research as possible in the future.

## Author contributions

**Conceptualization:** Xinru Liu, Yu Dai.

**Formal analysis:** Xinru Liu, Yilan Wang.

**Funding acquisition:** Quan Wen.

**Investigation:** Qing Ye, Yiming Sun, Jie Yang.

**Methodology:** Xinru Liu, Yilan Wang.

**Writing – original draft:** Xinru Liu.

**Writing – review & editing:** Yu Dai, Quan Wen.
